# F143. PREDICTORS OF RELAPSE: PATIENT, DISEASE, COGNITIVE, AND FUNCTIONAL CHARACTERISTICS WITH COMT GENE VAL158MET POLYMORPHISM IN A 2-YEAR FOLLOW-UP

**DOI:** 10.1093/schbul/sby017.674

**Published:** 2018-04-01

**Authors:** Anzalee Khan, Jean-Pierre Lindenmayer, Isidora Ljuri, Veronica Ozog, Amod Thanju

**Affiliations:** 1 Nathan S. Kline Institute for Psychiatric Research; 2 New York University; 3 Teachers College Columbia University

## Abstract

**Background:**

Schizophrenia is a severe and chronic mental illness characterized by continual relapses that may require hospitalization, changes in medications, arrests, emergency room hospitalizations, self-harm or suicidal behavior. Research has shown that costs associated with treatment received following relapse may constitute the largest share of treatment costs psychiatric illnesses. Although, demographic and clinical characteristics associated with relapse have been examined in previous research, information about potential predictors of relapse are limited. The aim of this study was to evaluate the effect of patient and disease characteristics, cognitive, functioning, and COMT gene polymorphism (rs4680) on relapse during 2-year following completion of an inpatient rehabilitation and cognitive treatment.

**Methods:**

Data were taken from a COMT genotype and response to cognitive remediation study of schizophrenia in the United States conducted between 07/2005 and 10/2015 for inpatients with schizophrenia who were also participating in psychiatric rehabilitation. Patients with and without relapse 2 years following completion of the study were compared on clinical, demographic, cognitive, functional and COMT genotype characteristics. The COMT gene rs4680 polymorphism was genotyped using a DNA sequence detection system. Relapse or events identified as treatment failures include: arrest, psychiatric re-hospitalization, suicide, discontinuation of antipsychotic treatment due to inadequate efficacy, treatment supplementation with another antipsychotic due to inadequate efficacy, discontinuation of antipsychotic treatment due to safety or tolerability, or increase in the level of psychiatric services. Baseline (end of study, start of 2-year follow-up) predictors of subsequent relapse were also assessed. Univariate Analsyis and Cox’s regression was used to examine the effect of potential predictors on outcome.

**Results:**

Of 140 subjects with eligible data, 91 (65.00%) relapsed during the 2-year follow-up period. Patients who relapsed were younger (< 45 years), higher number of previous hospitalizations, shorter chronicity of illness (< 10 years), PANSS baseline score of >4 on the core PANSS items (conceptual disorganization, hallucinatory behavior, suspiciousness, unusual thought content), higher negative symptom factor, substance use, PSP score of < 60 and lower MCCB composite T score (> 2 SD below the mean). Univariate analysis shows that COMT rs4680 gene variants were different between relapse and stable groups. The COMT rs4680 gene had an interaction with PANSS baseline core item scores and MCCB composite score. Number of previous antipsychotic trials did not predict relapse.

**Discussion:**

There is a high relapse rate within 2 years in chronic schizophrenia. Behavioral symptoms, aided by genetic and environmental factors common to this population (homelessness, unemployment, and social isolation) frequently lead to treatment failure. Knowing potential triggers of relapse can help in developing resources for this population to reduce treatment failures and associated costs.

